# A qualitative study of the sources of chronic obstructive pulmonary disease-related emotional distress

**DOI:** 10.1177/14799731231163873

**Published:** 2023-03-10

**Authors:** Diana Zanolari, Daniela Händler-Schuster, Christian Clarenbach, Gabriela Schmid-Mohler

**Affiliations:** 1 School of Health Sciences, Institute of Nursing, Zurich University of Applied Sciences, Winterthur, Switzerland; 2 Department Nursing Science and Gerontology, Institute of Nursing, Private University of Health Sciences Medical Informatics and Technology, Hall in Tyrol, Austria; 3 Te Kura Tapuhi Hauora, The School of Nursing, Midwifery, and Health Practice at Victoria University of Wellington Te Herenga Waka, Victoria University of Wellington, Wellington, New Zealand; 4 Department of Pulmonology, 27243 University Hospital Zurich, Zurich, Switzerland; 5 Centre of Clinical Nursing Science, 27243 University Hospital Zurich, Zurich, Switzerland

**Keywords:** Psychological distress, chronic obstructive pulmonary disease, qualitative research, anxiety, depression, self-management

## Abstract

**Objective:**

The aim of this study is to identify the sources of illness-related emotional distress from the perspective of individuals living with mild to severe chronic obstructive pulmonary disease (COPD).

**Methods:**

A qualitative study design with purposive sampling was applied at a Swiss University Hospital. Eleven interviews were conducted with individuals who suffered from COPD. To analyze data, framework analysis was used, guided by the recently presented model of illness-related emotional distress.

**Results:**

Six main sources for COPD-related emotional distress were identified: physical symptoms, treatment, restricted mobility, restricted social participation, unpredictability of disease course and COPD as stigmatizing disease. Additionally, life events, multimorbidity and living situation were found to be sources of non-COPD-related distress. Negative emotions ranged from anger, sadness, and frustration to desperation giving rise to the desire to die. Although most patients experience emotional distress regardless of the severity of COPD, the sources of distress appear to have an individual manifestation.

**Discussion:**

There is a need for a careful assessment of emotional distress among patients with COPD at all stages of the disease to provide patient-tailored interventions.

## Introduction

Chronic obstructive pulmonary disease (COPD) is an incurable disease, and its illness trajectory is characterized by an inexorable decline of lung function frequently accompanied by acute exacerbations.^1^ COPD brings with it high emotional distress,^2^ and increased prevalence of anxiety and depression compared to the general population.^3^ While patients living with COPD report various sources of emotional distress, high symptom burden due to breathlessness, coughing, fatigue, insomnia or weight is a major cause.^4^ Other sources of distress are hospitalization or fear of the future.^5^ Additionally, dependence on family members and role changes may engender guilt and frustration.^6^

Illness-related emotional distress has been found to be an important and independent factor for insufficient self-management leading to worse outcomes in other chronic conditions: In patients with diabetes illness-related emotional distress is known to negatively affect self-management resulting in higher glycated hemoglobin levels.^7^ Cancer-related emotional distress appears to be associated with significantly poorer adherence to chemotherapy^8^ Furthermore, several studies indicate that such illness-related emotional distress might also have a negative impact on self-management, reporting a decreased adherence to treatment for COPD.^9,10^ Several studies show illness-related emotional distress having a similarly negative impact on patients with COPD. For persons with COPD, illness-related emotional distress can result in poorer health outcomes such as self-reported functional limitations, poorer exercise tolerance, higher frequency of acute exacerbations and an increased length of hospital stay.^3,4,6,11^

Given the high prevalence of emotional distress in COPD and the negative impact of illness-related emotional distress, a careful assessment of emotional distress is needed in order to improve care of COPD patients.^12^ However, despite the importance of illness-related emotional distress in chronic conditions and self-management, to date there is no instrument to assess illness-related emotional distress in COPD nor is there any systematic knowledge of the causes of illness-related emotional distress in COPD.

The aim of this study was to identify the sources of illness-related distress from the perspective of individuals with COPD at all stages of the disease. It provides the basis for the development of a patient-reported experience measurement to assess the level and the sources of COPD-related emotional distress and provide patient-tailored interventions.

## Methods

### Study design

A qualitative research design using framework analysis was applied, which was developed by social researchers in the UK as an approach to analyzing qualitative data for policy research.^13^ The rule-based analysis was oriented towards mapping the lifeworld of the participants. This was done by transcribing the interviews and, in addition, by constantly comparing the material. For this purpose, a coding system was developed to make it possible to link the codes to overarching categories. The aim of this process was to achieve a maximum of objectivity in the evaluation.^14^ Ethics approval was granted by the Ethics Committee of Zurich, Switzerland (reference number: 2018-01455). Informed consent forms were signed by all participants.

As the theoretical framework to guide the data collection and analysis, the recently presented model for illness-related emotional distress for chronic respiratory disease was used, which is based on a systematic literature review and synthesis of five symptom management models.^15^ The model defines illness-related emotional distress as interaction between bodily symptom distress, treatment distress, distress due to restrictions in daily life roles and distress due to unpredictability. In addition to the level of emotional distress, the model describes individual’s goals and self-efficacy convictions as key drivers for self-management decisions in chronic respiratory diseases.

### Sampling and setting

The study was conducted between September 2018 and May 2019 at the Department of Pulmonology at University Hospital Zurich (USZ). There, individuals suffering from mild to severe COPD are treated both as outpatients and inpatients.

Inclusion criteria were a medical diagnosis of COPD and in- or outpatient treatment at USZ in the prior 12 months. Exclusion criteria were an inability to read or speak German or cognitive impairments.

Purposive sampling was applied to achieve maximum variation. A selection of clinical features that can lead to increased mortality were used as sampling criteria, i.e., global initiative for chronic obstructive lung disease (GOLD) stage (COPD 1–4), sex, age, BMI, use of oxygen at rest and the number of exacerbations in the last 12 months.^16,17^

### Recruitment and participants

As shown in Table 1, patient data relevant for the purposive sampling were extracted by the first author from USZ`s electronic medical history database in September 2018. Based on experience, a sample size of 10–12 patients was targeted and a decline rate of 50% (half) to 33% (one third) was assumed. Thus, a sample of 18 patients were chosen in which the purposive sampling criteria were equally distributed (25% for GOLD criteria and 50% for all other criteria). Those patients were informed about the study via a letter sent by post. A stamped, pre-addressed envelope was enclosed to return the signed informed consent form. One week later the first author called the patient to answer open questions. With patients who were willing to provide written informed consent, a date for the interview was fixed. Of the 18 eligible patients, five were unreachable and two patients refused to participate due to time constraints.

**Table 1.** Criteria for purposive sampling.

Criteria for purposive sampling				
Age	<65		≥65	
Sex	Female		Male	
BMI	<18.5		≥18.5	
FEV1 in stable phase (GOLD stage)	≤30	31–50%	51–80%	≥81%
Use of oxygen at rest	0 –1.75 L/min		>1.75 L/min	
Exacerbations in the last year	0 severe AND/OR ≤1 moderate^a^		≥1severe AND/OR >1 moderate	

^a^severe: lead to hospitalization, moderate: not lead to hospitalization.

### Data collection

Semi-structured narrative in-depth interviews were conducted by the first author. The interview guide is shown in Table 2. To describe the participants in detail, demographic data, the COPD Assessment Tool (CAT) as well as the Modified Medical Research Council (mMRC) were used at the end of each interview.

**Table 2.** Interview guide.

Questions
1. What does it mean for you to live with COPD?
2. Can you tell me what unpleasant emotions you experience sometimes because of COPD in everyday life?
3. What do you mean: why are these emotions triggered?
4. You mentioned strains like XY. What feelings does this trigger in you?
5. What other aspects of COPD cause emotional distress?
6. Can you describe a situation in which you felt particularly distressed?

All interviews were audiotaped and transcribed verbatim. All interviews were conducted and transcribed by the first author who was working as a nurse at USZ. With the exception of one patient who knew the first author from prior hospitalizations, none of the participants was previously known to the first author. After 10 interviews, no substantial information was added, so data collection was stopped after the eleventh interview.

### Data analysis

The iterative data analysis followed the five stages of framework analysis according to Ritchie and Lewis.^13,14^ A mixed inductive and deductive approach was used to analyze the data. To manage and analyze the data, NVivo 10® software was used. The five stages are described in Table 3.

**Table 3.** Stages of the analysis.

*Stage 1 Familiarization.* To get familiar with the respective data sets, the transcripts were read multiple times by the first author and, when necessary, the audio tapes were listened to again
*Stage 2 Constructing an initial thematic framework.* *Inductive category development.* First, data from all interviews was analyzed inductively by line-to-line coding by the first author. Codes were summarized inductively into subcategories *Deductive category development.* Based on the model of emotional distress in respiratory disease,^15^ the four sources of emotional distress were used as an initial framework for the main categories. Namely: ‘bodily symptoms’, ‘treatment’, ‘predictability’ and ‘restriction in daily life roles’ *Integration of deductive and inductive category development.* The final theoretical framework was developed by constantly comparing and contrasting data with the deductively derived main categories and the inductively evolved subcategories. This was done by the first author, in regular discussions and consensus with the second and last author. As a result, ‘restriction in daily life roles’ was subdivided into two main categories: ‘restricted physical mobility’ and ‘restricted social participation’. COPD as stigmatizing disease was added as an additional main category because it evolved inductively from the data. This resulted in the final thematic framework as depicted in Table 5
*Stage 3 Indexing and sorting.* The data was labelled according to the thematic framework by the first author and also partly by the last author
*Stage 4 Data summary and display.* The framework matrix was built with the categories (main and subcategories) in columns and with the individuals in rows. Data was summarized per cell, accompanied by defining quotes from participants by the first author
*Stage 5 Abstraction and interpretation.* Data was compared and contrasted using purposive sampling criteria, e.g. comparing data between patients with different GOLD stages by the first author and in regular discussions with the last author

### Trustworthiness

To ensure confirmability and dependability, members of the research team met at least monthly to discuss the quality of the interviews as well as temporary results. Additionally, the first and the fourth authors coded two transcripts independently. With the exception of one code, all codes matched. The different coding was discussed and a consensus found.

## Results

### Sample characteristics

Eleven patients participated (7 females, aged 51–81 years, FEV1 (17%–96%) (Table 4). Of the interviews, seven took place at patients’ homes, three at a hospital and one in a rehabilitation clinic. The interviews lasted between 30 and 78 min.

**Table 4.** Participant characteristics.

Participant characteristics	*N* = 11
Age in years (median, range)	67 (51–81)
Gender female/male (number)	7/4
BMI kg/m^2^ (median, range)	21.5 (15.8–42)
FEV_1_ (median, range)	50 (17–96)
Use of oxygen (number)	
None	6
Up to 1.75 L/min	2
More than 1.75 L/min	3
Stage of COPD (number)	
COPD 1 A	2
COPD 1 B	1
COPD 2 A	1
COPD 2 D	1
COPD 3 D	3
COPD 4 B	1
COPD 4 D	2
MMRC score (median, range)	18 (2–27)
CAT score (median, range)	2 (0–4)
Exacerbations in the last year (median, range)	1 (0–6)
Marital status (number)	
Married	5
Divorced	3
Widowed	3
Living situation (number)	
Living alone	5
Living with spouse	5
Living with a child	1
Nationality (number)	
Swiss	9
Other	2
Highest level of education (number)	
Compulsory education	3
Apprenticeship	4
Higher apprenticeship/college	4
Working situation (number)	
Retired	6
Unemployed	1
Employed full-time	1
Employed part-time	1
Invalidity insurance pension	2

### Qualitative results

Patients reported six main sources of COPD-related emotional distress, namely, physical symptoms, treatment, restricted physical mobility, restricted social participation, unpredictability of disease course and stigma. In addition, three influencing factors emerged: life events, living situation and multi-morbidity. The main and subcategories are displayed in Table 5.

**Table 5.** Categories.

Main category	Subcategory
Physical symptoms	Breathlessness
	Cough/mucus
	Fatigue
	Pain
	Erectile dysfunction
	Incontinence
Treatment	Oxygen therapy
	Recommendation for smoking cessation
	Relationship with the treatment team
	Lack of benefit from medications
Restricted physical mobility	Enormous physical and mental effort
	Uncontrollable circumstances outside the home
	Revealing of illness
Restricted social participation	Loss of daily contacts
	Difficulties participating in social activities
	Difficulties participating in conversations
Unpredictability of disease course	Inability to plan the future life
	Uncertainty in relationships
COPD as stigmatizing disease	Failure in the past
	Label of “Brought about their own illness”
Non-COPD-related distress	Multimorbidity
	Live events
	Living situation

**Figure 1.**
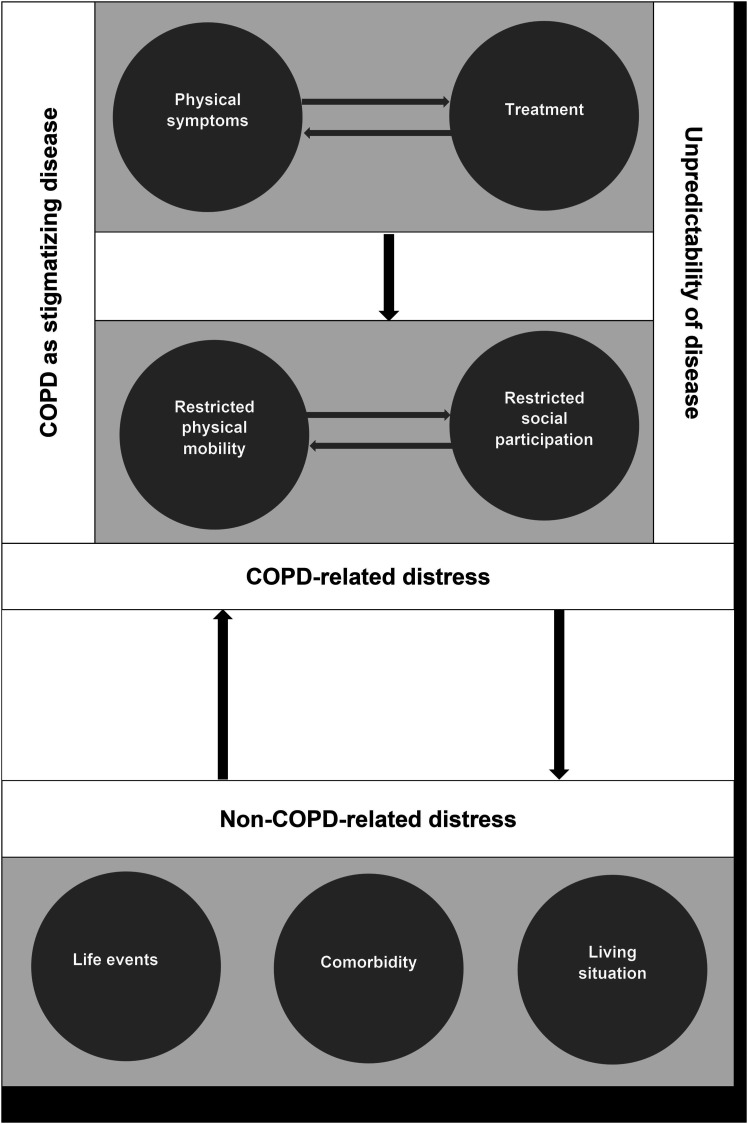
Sources of emotional distress and their interaction.

Emotional distress arose due to an interaction of all of these sources. The basis of emotional distress is formed by physical symptoms and treatment. The two in combination, especially breathlessness and oxygen therapy, lead directly to restricted physical mobility and are contributing factors for restricted social participation. Distress due to the unpredictability of disease course and COPD as a stigmatizing disease result from the overall experience of COPD (Figure 1).

#### Physical symptoms

Physical symptoms led to emotional distress when they are uncontrollable and not able to be explained. *Breathlessness* was the leading symptom, followed by *coughing and mucus, fatigue, pain, erectile dysfunction* and *incontinence*.

For the majority, *breathlessness* was a permanent and very stressful physical symptom that dominates everyday life and thoughts. As a consequence, participants experienced despair, powerlessness and unwillingness to live.

“(…) The first thought is about breathing and the last thought is about breathing (…) that's already really stressful (…) I have to tell you honestly, sometimes I think: "I’ve had enough". No, no, I can’t go on anymore (...) It's too much for me (…) Sometimes it makes me feel desperate. To the extent that I don't want to live anymore (…)”

Emma, COPD GOLD 2 D

*Coughing* was experienced as disruptive for all participants. Patients with COPD three and four experienced coughing combined with feelings of despair and helplessness, because episodes can last for hours. For patients with COPD GOLD Stage 1 and 2, coughing may have been the only sign of COPD. Coughing was described as distressing because it interfered with everyday activities such as having a conversation or sleeping.

Breathlessness and coughing caused *fatigue.* Furthermore, fatigue was reported as a source of distress by one patient with COPD 2 because the source of the symptom was not clear to her: It was present in the morning despite of having had enough sleep.

*Pain* was mentioned by nine participants, but only three participants spoke about pain related to COPD. For one patient with mild COPD, who underwent an operation due to pneumothorax, the pain was not so distressing. She was told that it might last for 6 months which helped her cope with it. In contrast, another patient who always felt chest pain when he experienced breathlessness described the pain as more distressing than the breathlessness itself.

One patient with COPD GOLD 3 experienced *erectile dysfunction* as burdensome because he assumed that the reason lies in his masculinity. He experienced relief after his family doctor had explained the connection with COPD and suggested the treatment with medication.

Three patients, one of them male, spoke of *incontinence* due to COPD and how highly distressing it was because of its unpredictability and negative impact on daily activities. They either did not reach the toilet in time or they lost urine during coughing. These situations were accompanied by feelings of shame and anger.

#### Treatment

Each of the participants described at least one therapeutic element as a source of emotional distress. Distressing elements were *oxygen therapy (CIPAP, BIPAP included), the recommendation for smoking cessation*, *relationship with the treatment team and lack of benefit from medications*. Participants with COPD 1 and 2 described increased emotional distress in relation to smoking cessation and treatment teams. Participants with COPD 3 and 4 named oxygen therapy as the strongest trigger for various negative emotions.

Two male patients in need of *oxygen therapy* in the form of BIPAP or CIPAP emphasized that despite having to adjust to the noise and uncomfortable mask initially, they did not feel emotionally distressed because it had no impact on their daily activities. This contrasts strongly with patients who needed continuous oxygen therapy COT via nasal cannula. They felt restricted in mobility due to the weight of the device, the shortness of the tube and the device’s lack of practicability.

For all patients who had not yet quit smoking, the *recommendation for smoking cessation* triggered feelings of anger. On the one hand, they had not stopped yet because they did not see the point in it, had no motivation or had already made several frustrating attempts with distressing adverse effects such as stomach pain. On the other hand, smoking cessation was experienced as a continually recurring topic mentioned by doctors.

The following were mentioned as a source of distress impeding the *relationship with the treatment team* and leading to considering a change of physician: a lack of time invested by the team and a lack of continuity, insufficient information about treatment options and COPD in general and not being taken seriously, particularly by pneumologists and general practitioners.

Two of the eleven participants, both with moderate COPD, did not have to take medication for COPD. Patients with severe and very severe COPD who had to take pills and inhale regularly all described this as part of their daily routine and not as distressing. *Medication* triggered negative feelings only when it *did not bring relief* from symptoms.

#### Restricted physical mobility

Participants with COPD 3, 4 and one patient with COPD 2 emphasized that their radius of movement got smaller as their disease progressed. For patients with a high symptom burden and oxygen therapy, there were three hindrances contributing to restricted physical mobility: *enormous physical and mental effort*, uncontrollable circumstances outside the home and *looking obviously ill* in front of others. All three barriers again led to negative emotions as they contributed to the fact that patients eventually became housebound. In contrast, participants with COPD 1 and 2 who did not suffer from any symptoms and did not need oxygen therapy pointed out that COPD did not cause them distress because they experienced no limitations in their daily activities.

Patients with COPD 3 and 4 stated that COPD affected all areas of life because everything entailed an *enormous physical and mental effort*. Even normal things such as taking a shower or going downstairs to the cellar took physical effort that they are sometimes incapable of making. Due to breathlessness and dependence on oxygen therapy in particular, the mobility of patients with severe and very severe COPD (and for one patient with moderate COPD), was restricted. As soon as the patients moved they were confronted with their physical limitations. This resulted in reduced scope of movement which eventually led to an inability to take care of themselves or to carry out daily activities like vacuum cleaning, lifting things, cleaning the house and carrying out daily morning routines like personal hygiene. These physical restrictions triggered intense negative emotions such as anger, sadness and frustration. This enormous physical effort also entailed a mental effort as patients were obliged to constantly assess whether the (physical) effort was in fact worth it. This continuously distressing evaluation process intensified as the disease progresses. The incessant uncertainty of what their health condition will be like from one moment to the next and the unpredictability of symptom severity may lead to continual anxiety during daily activities or when patients leave the house.

In addition, *uncontrollable circumstances when outside the home,* including strong smells, crowded places, or narrow spaces are described as very distressing because they can cause breathlessness and general discomfort. Several patients stated that they never knew whether they might unexpectedly have to return back home because of sudden breathlessness. This anxiety persists in every kind of daily activity whether it happens in public or at home. One woman points out:

“(...) somehow everything scares you a little. Sometimes, if you`re not feeling well enough to go outside and you still leave your apartment (...) you think: (…) Will I be able to return back home on my own?’”

Maria, COPD GOLD 3 D

When being physically active, patients had to stand still, use an oxygen device/nasal cannula, or to breathe heavily and visibly. Physical activity *revealed the illness* in front of others, which made it into another source of distress, triggering feelings of shame and anger. Participants explained that they did not want others to see them being ill or suffering.

#### Restricted social participation

As a result, their participation in social life with activities such as pursuing a hobby, meeting other people or going to work grew increasingly more difficult. At this point in the interview, some participants started to cry and expressed strong negative emotions ranging from anger, disappointment and frustration to depression. Eventually, the lives of some participants, especially the older ones, were completely restricted to their own homes. Three sources of emotional distress were found regarding restricted social participation: *losing daily contacts*, *difficulty participating in social activities* and *difficulty participating in conversations.*

Due to the increasing inability to leave their apartment, it grew more *difficult to maintain social contact*s *on a day-to-day basis*, leading to feelings of being home bound and being forgotten by the outside world.

*Not being able to participate in social activities* such as hiking, cycling, skiing, dancing, attending a play or concert or going to a restaurant with others triggered feelings of loneliness, worthlessness and frustration.

“(…) when you can’t go out and do anything, you get depressed. If they call and ask you to come to the cinema or the theatre and you have to say: I can’t, you’re bound to get depressed (…) and no one visits you.”

Ruth, COPD GOLD 4 D

Some patients realized at a certain point that they were no longer able to keep up with others and that meeting people spontaneously or keeping long-term appointments was challenging. Consequently, they only left the house with friends and family members who supported them and were willing to adapt activities to their state of health. Being dependent on other people’s consideration resulted in feelings of being a burden or of experiencing themselves as “a disabled person” when, for example, they had to ask others to reduce their pace. Patients in a relationship pointed out that their partners were also subject to all of these restrictions. They emphasized that they did not want to be a burden and therefore let their partners pursue their hobbies on their own, even if they feared drifting apart.

Another source of distress was the d*eclining ability to have longer conversations*, be it on the telephone or face-to-face. For some patients, as the disease progressed it became more and more difficult to speak while walking or having a meal with friends due to breathlessness.

#### Unpredictability of disease course

Unpredictability of disease course was for most patients a source of strong emotional distress, because knowing that the disease and the limitations will get worse made it *difficult to plan the future*. This triggered feelings of anxiety, sadness and uncertainty:

“It turns your existence upside down (...) You had a plan for the time after your retirement. And that plan won’t work anymore. You have to turn everything around and you have to change it (the life plan) completely (...).”

Kevin, COPD GOLD 4 B

Uncertainty over the course of the disease also entailed *uncertainty regarding how existing partnerships would develop* in future or whether life made any sense at all. Four female participants talked about how they sometimes thought they would be better off dead because they no longer wanted to live like this or endure the disease progression. Two participants revealed that they were planning to rely on euthanasia.

#### COPD as a stigmatizing disease

All of the patients who had previously been smokers reported the *failure of not having quit earlier*, connected with feelings of guilt and shame. Moreover, some participants, regardless of severity of COPD, reported feeling that they have been *labelled as having “brought about their own illness”*, namely by health professionals, relatives, friends, but also by society in general. Most participants acknowledged smoking as a major cause of COPD. At the same time, they all asserted that reasons other than smoking have also contributed to their acquiring *COPD*, for example, exposure at workplaces and air pollution, but that nobody had taken that seriously. This was a potential source of anger. Both patients suffering from antitrypsine deficiency strongly emphasized that they had never smoked. In addition, they stressed that they did not want to “give a false impression” or of “having screwed up by themselves”

#### Non-COPD-related distress

Participants reported some sources of non-COPD-related distress that can interact negatively with COPD-related distress. Namely, *multimorbidity*, *life events* and *living situation*.

For older patients with COPD 3 and 4 who suffered from *multimorbidity* such as heart failure or osteoporosis, COPD was accompanied by the most limiting symptoms. However, they related that the situation, which involved other health problems, was also linked to emotional distress. In contrast, two patients with COPD 1 lung cancer who had had myocardial infarctions regarded COPD as a “side issue” and therefore less of a burden than other health problems.

Stressful life situations such as disputes at work, in the neighbourhood, within the family or even the death of a relative were described as challenging. Such events intensified breathlessness. Patients reported that, due to COPD, they were no longer as resilient as before when confronted with critical *life events*.

The *living situation* and the general environmental situation of patients could be distressing. Living alone, on an upper floor without a lift, or problems accessing shopping facilities could also reinforce the COPD - related distress.

## Discussion

This study aimed to identify sources of emotional distress arising from living with COPD from patients’ personal perspectives. To our knowledge, this is the first study with the aim of finding sources of distress. Physical symptoms, treatment, restricted physical mobility, restricted social participation, COPD as a stigmatizing disease and unpredictability of the disease course were identified as major sources of COPD-related emotional distress. Additionally, three significant triggers of non-COPD-related emotional distress, namely multimorbidity, life events and living situation were found to have the potential to interact negatively with COPD-related emotional distress.

In line with the existing body of research,^5^ shortness of breath, cough and phlegm, as well as pain were identified as common and distressing symptoms in patients with COPD. Although several quantitative studies indicate that sexual dysfunction and urinary incontinence are very common in both women and men with COPD,^18,19^ our study underlines their distressing aspect, particularly in younger patients. It highlights the need to systematically screen and discuss these intimate symptoms with patients.

A restricted social life has been identified as distressing in other studies,^20^ with the reaction of our participants indicating that it may be the strongest contributing source of COPD-related emotional distress This highlights the need for strategies to be developed regarding COPD patients that might increase their feelings of security when leaving the house. This can include calling in a companion service, calling a taxi, creating an action plan and involving relatives.^21^ In addition, peer support and interaction with nurses was perceived as helpful to diminish the feeling of isolation.^22^ Patients who smoked reported experiencing stigmatization by health professionals, friends and family, as well as society in general. This is in line with other findings that report stigma as being an additional contributor to social isolation, feelings of loneliness and guilt and an influencing factor on medication-adherence and help-seeking.^23,24^

Notably, in the present study the relationship to health professionals was described as an important source of distress. Unmet needs and a lack of trust in the treatment team have been reported as significantly impeding disease management in patients with COPD.^25^ Our study highlights the necessity of a coordinated care model for patients with COPD, with a focus on continuity in care as a perquisite to building a trustful relationship, which in turn has the potential to impact outcomes.^22,26^ This would facilitate the approach when addressing sensitive topics such as smoking cessation, urinary incontinence, sexual dysfunction, stigmatization, and social isolation as well as the consideration of euthanasia.

By contrasting our study to the conceptual model of emotional distress,^15^ our study confirmed the theoretical framework that COPD-related emotional distress arose from an interaction of all six sources. New was the identification of stigma as an additional source, which in turn influences social interaction and self-image. Interestingly, this is the main difference to the model, where stigmatizing disease was not identified as a source of distress. One explanation may be that the model development also included studies with Cystic Fibrosis (CF). As CF is an inherited disease, the aspect of a “self-inflicted disease” might be omitted and thus perceived stigmatization might not be an important and distressing issue.

This study highlights the need for the current practice to be supplemented by the assessment and treatment of COPD-related emotional distress. Furthermore, all patients currently experiencing emotional distress as well as those without any signs should be regularly assessed to recognize the presence of sources of distress at an early stage of the disease. An assessment should be developed to assess sources of distress in these patients. The present study serves as the basis for the development of such a questionnaire and the results should be evaluated and confirmed by a quantitative study. In addition, the interaction of the different sources of COPD-related emotional distress and their impact on adherence and decision-making regarding self-management needs further investigation.

This study was conducted pre-COVID, prompting the question of whether the pandemic would have influenced the results. COPD patients were more vulnerable to COVID-19 and at risk for complications and poorer outcomes.^27^ Patients who were aware of being at high-risk, redrew socially in order to avoid infection, especially in the early stages of the pandemic. This led to increased levels of anxiety and feelings of loneliness.^28^ This indicates that patients with COPD may have experienced higher levels of illness-related emotional distress during the pandemic, but that the sources were the same as found in this research, namely unpredictability and restricted social participation.

This study has some strengths and limitations: Comparing and contrasting the patients’ stories using Framework Analysis allowed the depiction of a common story. The purposive sampling resulted in a heterogeneous group that allowed a broad description of patients’ experiences. However, there are also limitations that must be mentioned. First, participants were recruited from a single hospital and included only two patients with a migration background. Possibly patients from other geographical and cultural backgrounds may have described other sources of distress.

No additional sources of distress were detected after the tenth interview. In addition to redundancy in the reported sources of distress, a conceptual depth of how the sources were reported by patients in different stages of the disease was observed. This is at a rather early stage, but not surprising because the research question had a clear focus, was discovery-oriented, and the research was embedded in a pre-existing theoretical framework.^29^

## Conclusion

Living with COPD is associated with emotional distress and the sources are manifold, ranging from burdensome symptoms or treatment to restrictions in daily life and social participation, as well as stigma and unpredictability of the disease. Further, emotional distress seems to affect self-management in COPD, highlighting the importance of the concept of illness-related emotional distress in COPD. The next steps would be the development of a patient-reported experience measure for assessing illness-related emotional distress to clarify its role in self-management and patient outcomes.
